# Recombinant adenoviral vaccine encoding the spike 1 subunit of the *Middle East Respiratory Syndrome Coronavirus* elicits strong humoral and cellular immune responses in mice

**DOI:** 10.14202/vetworld.2019.1554-1562

**Published:** 2019-10-11

**Authors:** Mustafa Ababneh, Mu’men Alrwashdeh, Mohammad Khalifeh

**Affiliations:** Department of Basic Medical Veterinary Sciences, Jordan University of Science and Technology, P.O. Box 3030, Irbid 22110, Jordan

**Keywords:** coronavirus, *Middle East respiratory syndrome*, recombinant vaccine, spike protein

## Abstract

**Background and Aim:**

*Middle East respiratory syndrome coronavirus* (MERS-CoV) has rapidly spread throughout the Middle East since its discovery in 2012. The virus poses a significant global public health threat with potentially devastating effects. In this study, a recombinant adenoviral-based vaccine encoding the spike 1 (S1) subunit of the MERS-CoV genome was constructed, and its humoral, and cellular immune responses were evaluated in mice.

**Materials and Methods:**

Mice were immunized initially by intramuscular injection and boosted 3 weeks later by intranasal application. Expression of the S1 protein in the lungs and kidneys was detected using conventional polymerase chain reaction (PCR) and immunohistochemistry (IHC) targeting specific regions within the S1 subunit at weeks 3, 4, 5, and 6 after the first vaccination. Antigen-specific humoral and cellular immune responses were evaluated in serum and in cell culture following *in vitro* stimulation with a specific 9-mer epitope within the S1 protein (CYSSLILDY).

**Results:**

S1 protein expression was only detected by IHC in the kidneys of the Ad-MERS-S1 group at week 6 from first immunization, and in both lungs and kidneys of Ad-MERS-S1 group by conventional PCR at weeks 3 and 5 post-prime. The vaccine elicited a specific S1-immunoglobulin G antibody response, which was detected in the sera of the vaccinated mice at weeks 4 and 6 from the onset of the first immunization. There was a significant increase in the amount of Th1-related cytokines (interferon-γ and interleukin [IL] 12), and a significant decrease in the Th2-related cytokine IL-4 in splenocyte cell culture of the vaccinated group compared with the control groups.

**Conclusion:**

The results of this study suggest that this recombinant adenovirus vaccine encoding the S1 subunit of MERS-CoV elicits potentially protective antigen-specific humoral and cellular immune responses in mice. This study demonstrates a promising vaccine for the control and/or prevention of MERS-CoV infection in humans.

## Introduction

*Middle East respiratory syndrome coronavirus* (MERS-CoV) is a newly emerging human coronavirus that was discovered in 2012 in a 60-year-old Saudi Arabian man [1]. Following its discovery, many cases were identified in different regions of the Arabian Peninsula and worldwide thereafter [2,3]. The most recent outbreak occurred in June 2015 in South Korea and was linked to a South Korean man who had recently traveled to the Middle East [3]. The infection then rapidly spread to 26 persons through close contact in a hospital. Within a few months, many cases (n=186) were reported in hospitalized and non-hospitalized persons in South Korea [3]. The disease showed a high mortality rate that reached up to 40%, which was higher than that of the severe acute respiratory syndrome coronavirus (SARS-CoV) outbreak in 2002-2003 (10%) [4].

Coronaviruses belong to the subfamily Coronavirinae within the family *Coronaviridae* of the order *Nidovirales* [5]. Five human coronaviruses were identified (229E, OC43, NL63, HKU1, and SARS-CoV) before MERS-CoV. Lineage C of betacoronaviruses includes bat coronaviruses, which give a primitive impression regarding the origin of the virus [6]. The detection of MERS-CoV and its neutralizing antibodies in the sera of dromedary camels has shed light on the role of the camel as a possible animal reservoir, which may transmit the virus to humans [7-10]. Indeed, researchers isolated the same MERS-CoV strain from both a camel “in a barn” and its infected owner in Saudi Arabia, thus providing further evidence of the potential airborne and direct contact transmission of the virus between camels and humans [11].

There have been several attempts to develop a protective vaccine against MERS-CoV [12-23]. Researchers around the world are focused on the spike protein as the main target for vaccine development against MERS-CoV. The spike protein of MERS-CoV attaches to the host dipeptidyl peptidase-4 (DPP4) receptor, which is expressed on several types of human cells [24]. Many studies published since 2012 suggesting vaccine models were constructed based on the spike protein and its receptor-binding domain (RBD) region to elicit a strong and protective immune response *in vitro* and *in vivo* [25]. Recombinant adenoviral vector vaccines are one of the most effective vaccines and showed interesting results during SARS-CoV outbreaks [12,26,27]. Since 2013, several studies were published, in which different viral vectors (e.g., adenoviruses and vaccinia virus) were used to develop recombinant vaccine candidates based on full spike gene or part of it and tested their ability to produce protective immunity against MERS-CoV infection [13-23]. However, further investigations are needed on these suggested vaccines including testing their ability to elicit neutralizing antibodies in different animal models, stimulation of both innate and adaptive immune responses and their corresponding cytokine and chemokine profiles, distribution within the host, and their safety and duration profiles.

In this study, a recombinant adenoviral-based vaccine encoding the spike 1 (S1) subunit of the MERS-CoV genome was constructed, and its humoral, and cellular immune responses were evaluated in mice.

## Materials and Methods

### Ethical approval

All procedures performed in this study were approved by the Jordan University of Science and Technology Animal Use and Care Committee.

### Construction of the recombinant vaccine

The recombinant human adenovirus type 5 (dE1/E3) vaccine was designed to express the S1 domain of the spike protein of MERS-CoV isolate Hu/Jordan-20140010168/2014 (GenBank accession number KT861627) under the control of the cytomegalovirus (CMV) promoter. This vaccine was constructed in the Laboratories of Vector Biolabs (The Gene Delivery Company, PA, USA) [28].

### Experimental design

Six to eight week-old male BALB/c mice (n=168) were purchased from Animal House at Jordan University of Science and Technology (Irbid-Jordan) and distributed into three groups in a replicate manner (56 each). Animals in the first group were immunized with the Ad-CMV-MERS-CoV-Spike1 “Ad-MERS-S1.” Animals in the second group received the vector-only “Ad-CMV,” and animals in the last group served as a control and received 1× phosphate-buffered saline (PBS). For the first immunization, the Ad-MERS-S1 vaccine was injected intramuscularly at a dose of 0.1 ml containing 1×10^7^ PFU/dose/mouse. The same volume of Ad-CMV or PBS was used for the other two groups. The booster dose was carried out 3 weeks later by intranasal route at a dose of 0.1 ml containing 1×10^7^ PFU/dose/mouse. All experimental procedures used in this study were approved by the Animal Care and Use Committee at the Faculty of Veterinary Medicine (Irbid-Jordan).

### Sample collection and processing

Lung, kidney, and spleen tissue samples were harvested from each mouse in all groups after 3, 4, 5, and 6 weeks from the first immunization. Parts of lung and kidneys were preserved in 10% formalin solution for 24-h, then embedded in paraffin blocks for immunohistochemical analysis, while the other parts were stored at −80°C for polymerase chain reaction (PCR) analysis. Spleen tissues were harvested and stored at −80°C for cell culture analysis. Blood samples were drawn through facial vein route, left 30 min at room temperature, and centrifuged at 2000 rpm for 20 min. Collected sera were stored at −20°C for subsequent analysis.

### Detection of S1 gene expression by heminested PCR

To study the distribution of the recombinant vaccine, total of 50-100 mg of lung and kidney tissues at weeks 3 and 5 post-prime immunization was cut and homogenized in 1 ml of diethyl pyrocarbonate-treated water. Homogenized samples were then centrifuged at 800 rpm for 5 min, and 150 µl of the supernatant was used for the extraction. The extraction was performed using the Viral Gene-Spin RNA Extraction Kit (Intron Biotechnology, Korea) according to the manufacturer’s instructions. The extracted RNA was used for cDNA synthesis through a reverse transcription system (Promega, Madison, WI, USA) according to the manufacturer’s instructions. The cDNA was kept at −20°C until PCR analysis. The DNA template of the constructed vaccine was also purified and used as a positive control in this experiment.

Heminested PCR was carried out using specific primers targeting specific sequences within the S1 region, as shown in Table-1 [29]. The first PCR targeted a 1172 bp sequence. PCR mixture contained 0.4 µM of each primer and using 3 µl of the cDNA template. Touchdown PCR assay was programmed on the MyCycler thermal cycler (Bio-Rad Laboratories, Hercules, CA, USA) as the following conditions: 95°C for 10 min, followed by 10 cycles of 95°C for 35 s, 63-54°C for 40 s, and 72°C for 1 min and 15 s, then 35 cycles of 95°C for 40 s, 56°C for 40 s, 72°C for 1 min and 15 s, and a final extension step at 72°C for 5 min. Two microliters from the first PCR product were used as a template for the second (heminested) PCR to amplify the 1142 bp product following the first PCR conditions. The bands were visualized in 1.5% agarose gels using an ultra-violet transilluminator.

**Table 1:** Primer sequences for heminested PCR targeting the S1 segment.

Primer	Sequence (5’- 3’) and expected PCR product size	Reference
NM-SPIKE-F3	ACGTCAAACAGTTYGCTAATGG	[29]
NM-SPIKE-R1	GCTGTACTTAAGAGGCTTAGTAAT	
NM-SPIKE-R2	GTAAGGTTATGAGGAACAGTCGC	
1^st^ PCR (F3+R1)	1172 bp	
2^nd^ PCR (F3+R2)	1142 bp	

PCR=Polymerase chain reaction

### Immunohistochemistry (IHC)

Localization of S1 protein expression was investigated in both kidney and lung tissues from all groups at week 6 after the first vaccination. IHC using rabbit-specific HRP/DAB (ABC) detection IHC kit (Abcam, Cambridge, UK) was adapted, with using antinovel coronavirus spike protein S1 polyclonal antibodies (1:1000; Sino-Biological, PA, USA). A 5-µm thick section of formalin was fixed, and paraffin-embedded tissues were cut and loaded on specially coated slides. The prepared slides were put in an oven at 70°C for 30 min followed by 15 min in a xylene solution for deparaffinization. The sections were dried and surrounded by liquid blocker (Dako pen). The antigen retrieval (pretreatment) step was executed by immersing the sections in 1× reveal Decloaker reagent (Biocare Medical, Pacheco, CA, USA) and heating in a microwave several times for 30 min then cool at room temperature for 15-20 min. After that, staining procedure was carried out by following the manufacturer’s instructions of the ABC kit. To eliminate the effect of internal renal biotin, 0.05% avidin was applied (Sigma-Aldrich, St. Louis, MO, USA) only on kidney sections for 15 min at room temperature [30,31]. The immunoreactivity of the expressed S1 protein was visualized using a light microscope at different magnifications.

### Lymphocyte cell culture

Spleen tissues from each group were collected, pooled, and homogenized to obtain single-cell suspensions. The cells were centrifuged for 10 min at 800 rpm, and the pellet was re-suspended in Roswell Park Memorial Institute Medium (RPMI) (Gibco, Grand Island, NY, USA), supplemented with 10% fetal calf serum, 20 mM HEPES, 10 µg/ml penicillin/streptomycin, 2 mM L-glutamine, 50 µM 2-mercaptoethanol, and 500 ng/ml Amphiostat B (complete RPMI) [32]. The cell suspension was washed again by centrifugation as described earlier. Spleen cells were re-suspended in red blood cell lysis buffer (Tris-buffered NH_4_CL) to remove erythrocytes. Cells from each group (1×10^5^) were re-suspended in 1 ml of complete RPMI and plated in 24-well plates in triplicate in parallel, adjacent wells. The first set of samples was stimulated with 10 µg of specific peptide “epitope” CYSSLILDY per well. This antigen was defined as the highest potential T-cell epitope from a total of eight potential epitopes within the RBD region of the S1 subunit of the spike protein of MERS-CoV genome and was expected to display interactions with the maximum number of major histocompatibility complex (MHC) Class I molecules, and to show the highest peak in the B-cell antigenicity plot [33]. The second set of samples was stimulated with 50 µg of non-specific stimulant phytohemagglutinin to confirm the viability and productivity of the cultured cells. The third set of samples was not stimulated as a control set. Cells were incubated in round-bottom 24-well microtiter plates at 37°C in 5% CO_2_. The cultured media were collected after 96 h, and the samples were centrifuged for 20 min at 2000 rpm. The supernatants were stored at −20°C before cytokine analysis, while the pellets were stored at −80°C until real-time (RT)-PCR analysis.

### Humoral MERS-S1 immunoglobulin G (IgG) response in mice sera

Detection and measurement of specific MERS-S1 IgG were achieved using a semi-quantitative anti-MERS-CoV ELISA (IgG) (EUROIMMUN AG, Luebeck, Germany) with a slight modification using universal enzyme conjugate, anti-mouse IgG-conjugated HRP, to be able to detect mouse IgG antibodies instead of human IgG antibodies. Microtiter plates were pre-coated with purified S1 antigen of MERS-CoV. Mouse diluted serum samples (1:100) were incubated in each well from the three experimental groups (Ad-MERS-S1, Ad-CMV, and PBS) for 30 min at room temperature by following the manufacturer’s instructions. The color intensity was measured using an ELISA plate reader (BioTek, Winooski, VT, USA).

### Detection of mouse cytokines (interferon-γ [IFN-γ], interleukin [IL]-12, and IL-4) in cell culture supernatant

Cell culture supernatants from stimulated and corresponding non-stimulated samples of the three groups were tested for the level of mouse cytokines at weeks 4 and 6 post-prime using a quantitative sandwich enzyme immunoassay technique (Quantikine ELISA Mouse Cytokines Immunoassay, Minneapolis, MN, USA) according to the manufacturer’s instructions.

### Gene expression of mouse cytokines (IFN-γ and IL-4) in the cell culture pellet

Relative quantification of mouse IFN-γ and IL-4 was normalized to the housekeeping gene β-actin in the cell culture yield of stimulated groups. The collected pellets from cell culture were used to extract total RNA using the SV Total RNA Isolation System (Promega) according to the manufacturer’s instructions; this extraction kit has a DNase incubation step to eliminate any DNA in the sample. Isolated RNA (40 µl) was stored at −80°C before RT-PCR analysis. RNA concentration was adjusted to 10 ng/µl using TE buffer. Purified RNA (10 µl) was used as a template to produce 20 µl of cDNA using the reverse transcription system (Promega) kit. Relative RT-PCR assay was performed to determine the mRNA expression levels of mouse IFN-γ and IL-4 in the cell culture. The fold changes were calculated using the 2^−ΔΔCT^ method normalized to β-actin [34]. Specific primer sets were used, as shown in Table-2 [35]. Extracted RNA (2 µl) was used as a template in the PCR reaction mixture (20 µl), which was composed of 0.5 µM each of the forward and reverse primers, 10 µl of PowerUp SYPR Green Master Mix (Applied Biosystems, Foster City, CA, USA), and 6 µl of nuclease-free water. RT-PCR conditions were programmed in Rotor Gene-Q (Qiagen, Hilden, Germany) as follows: 50°C for 2 min, 95°C for 10 min, followed by 40 cycles of 95°C s, 60°C for 1 min, and a melting step at 55-99°C for 30 s.

**Table 2:** Mouse cytokines and β-actin primer sequences for real-time PCR.

Gene	Primer	Sequence (5’- 3’)	Reference
IFN-γ	Forward Reverse	TCAAGTGGCATAGATGTGGAAGAA TGGCTCTGCAGGATTTTCATG	[35]
IL-4	Forward Reverse	GGCATTTTGAACGAGGTCACA AGGACGTTTGGCACATCCAT	
β-actin	Forward Reverse	AGAGGGAAATCGTGCGTGAC CAATAGTGATGACCTGGCCGT	

PCR=Polymerase chain reaction, IFN-γ=Interferon-γ, IL=Interleukin

### Statistical analysis

Data for ELISA and fold changes were expressed at mean ± standard deviation. One-way ANOVA and t-test were used to compare different values in all groups using OpenEpi software (Emory University, USA). Parameter differences were considered statistically significant at p<0.05. Parameter differences were indicated by small letters labeled within each group; different letters indicated significant differences at p<0.05.

## Results

### Biodistribution and localization of S1 protein in mice tissues

**Figure-1:**
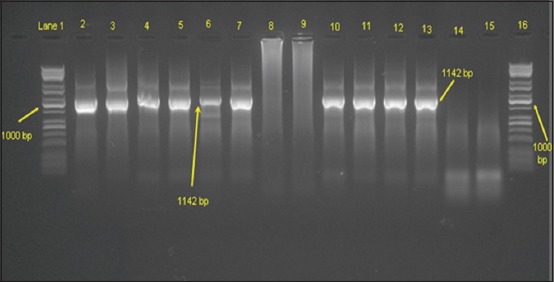
Electrophoresis (1.5% agarose gel) of *Middle East respiratory syndrome coronavirus*-S1 segment of the spike gene. Lanes 1 and 16, Kapa Universal DNA Ladder; lanes 2 and 3, vector vaccine extracted DNA (positive controls); lanes 4-7, positive samples from two animals in the vaccinated group at week 3 (lanes 4 and 6, lungs; lanes 5 and 7, kidneys); and lanes 8 and 9, negative control samples at 3 weeks collected from control groups. Lanes 8 and 9 are pooled tissues (lung and kidney) from the Ad-cytomegalovirus and phosphate-buffered saline groups, respectively. The same order for the samples collected at 5 weeks after vaccination is presented in lanes 10-15. Amplicon size is 1142 bp.

**Figure-2:**
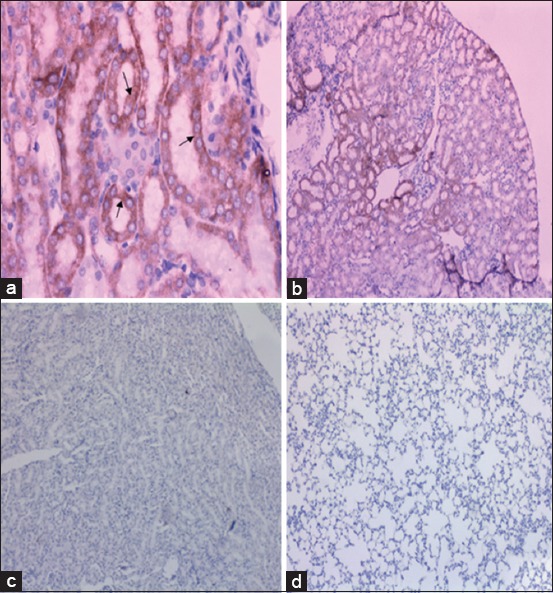
Immunolocalization of the S1 protein in mice tissues. (a and b) Immunoreactivity of the S1 protein in the proximal tubules of mouse kidney from the vaccinated group with Ad-*Middle East respiratory syndrome coronavirus*-S1 at week 6 post-vaccination (400×, 100×). (c) Proximal tubules of mouse kidney from the control group 6 weeks after the onset of treatment show no expression of the S1 protein (100×). (d) Lung tissues of vaccinated mice show no immunoreactivity to S1 protein (100×).

Distribution and expression of the S1 subunit of the MERS-CoV spike protein in mice tissues were detected at weeks 3 and 5 post first immunization in the kidneys and lungs of the vaccinated group but not in control groups using conventional PCR (Figure-1). Using IHC, S1 protein expression was detected in the cytoplasm of proximal tubule epithelium in the vaccinated mice at week 6 post first immunization but not in the control groups (Figure-2). S1 expression was not detected in lung tissues in either the vaccinated or the control groups (Figure-2).

### Detection of specific MERS-S1-IgG in mice sera

**Figure-3:**
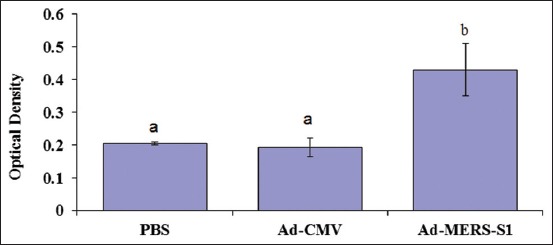
*Middle East respiratory syndrome coronavirus* (MERS)-specific serum immunoglobulin G (IgG) antibody response. A significant level of MERS-S1-specific IgG antibodies in the vaccinated group (n=18) was detected at weeks 4 and 6 post-prime. Different letters indicate significant differences at p<0.05.

There was a significant level (p<0.05) of MERS-S1 specific IgG antibodies detected in the mice sera of the vaccinated group at weeks 4 and 6 post-prime. The combined results of weeks 4 and 6 revealed that the IgG antibody response was significantly higher (p<0.05) in the vaccinated group than in the control groups (Figure-3).

### Effect of vaccination and antigen-specific stimulation on the production level of mouse cytokines *in vitro*

**Figure-4:**
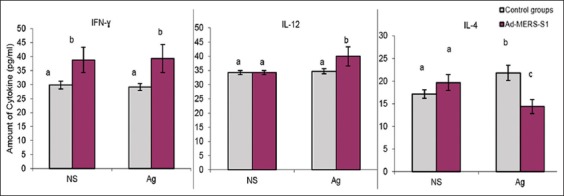
Effects of vaccination and antigen-specific stimulation on the production of mouse cytokines (interferon-γ [IFN-γ], interleukin 12 [IL-12], and IL-4) *in vitro*. Regardless of *in vitro* stimulation, production of IFN-γ was significantly (p<0.05) higher in the vaccinated group with Ad-Middle East respiratory syndrome (MERS)-S1 compared with the control groups (Ad-cytomegalovirus and phosphate-buffered saline) at all tested time points. *In vitro*, antigen-specific stimulation resulted in a significant upregulation of IL-12 production when compared with the antigen-stimulated and non-stimulated cell culture in the vaccinated group only. *In vitro* stimulation using the MERS-specific peptide “CYSSLILDY” resulted in a significant upregulation of IL-4 production in the control groups while the same antigen stimulation resulted in a significant decrease in IL-4 production in the vaccinated group. NS: Non-antigen stimulated, Ag: Antigen-stimulated. Different letters mean significantly different at p<0.05.

The level of mouse cytokines at weeks 4 and 6 after the first vaccination in the cell culture supernatants of stimulated and corresponding non-stimulated samples of the three groups was tested by sandwich ELISA assay. Mice cytokines (IFN-γ, IL-12, and IL-4) production in the Ad-CMV and PBS groups were combined and expressed collectively as one group (control groups) due to the non-significant differences shown across these time points between these two groups (Figure-4). Therefore, the levels of each cytokine at the two tested time points (weeks 4 and 6 after the first vaccination) were also combined and expressed as one unit.

Regardless of *in vitro* stimulation, production of IFN-γ was significantly (p<0.05) higher in the vaccinated group compared with the control groups (Ad-CMV and PBS) at all tested time points (Figure-4).

In terms of IL-12 production, *in vitro* stimulation with the MERS-specific peptide “CYSSLILDY” resulted in a significant (p<0.05) upregulation in production when compared with the antigen-stimulated and non-stimulated cell culture in the vaccinated group only (Figure-4).

Although there was no significant difference in IL-4 production between the control groups and the vaccinated group in non-stimulated cell culture, antigen-specific stimulation clearly revealed a higher production of this cytokine in the control groups above the vaccinated group level of production (p<0.05). In addition, *in vitro* stimulation with the MERS specific peptide “CYSSLILDY” resulted in a significant upregulation in this production of this cytokine in the control groups while the same antigen stimulation resulted in a significant decrease in IL-4 production in the vaccinated group (p<0.05) (Figure-4).

### Gene expression of mouse cytokines in the cell culture pellet

**Figure-5:**
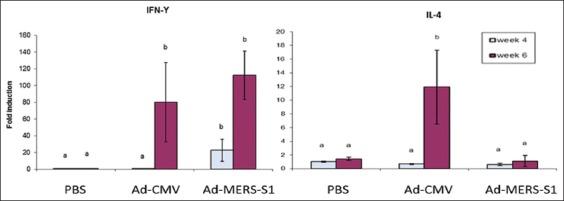
Interferon-γ (IFN-γ) and interleukin-4 (IL-4) gene expression normalized to β-actin at weeks 4 and 6 post-prime. At week 4, the fold change in IFN-γ gene expression was significantly higher in the Ad-Middle East respiratory syndrome (MERS)-S1 group than that of both control groups (Ad-cytomegalovirus [CMV] and phosphate-buffered saline). However, there was a prominent increase in IFN-γ gene expression at week 6 in both Ad-MERS-S1 and Ad-CMV groups compared to that in week 4. The only significant change in IL-4 gene expression was in the Ad-CMV group at week 6, while no significant changes were observed in the Ad-MERS-S1 group at any time point. Different letters indicate statistically significant differences at p<0.05.

At week 4 post first immunization, the fold change in IFN-γ gene expression was significantly higher than that in the control groups (Ad-CMV and PBS) (p<0.05). However, there was a prominent increase in IFN-γ gene expression at week 6 post-prime in both the vaccinated and Ad-CMV groups compared to that in week 4 (p<0.05). The expression of IFN-γ was slightly higher but not statistically significant in the vaccinated group compared to that in the Ad-CMV group at week 6 (p>0.05) (Figure-5).

The only significant change (p<0.05) in IL-4 gene expression was observed in the Ad-CMV group at week 6 (Figure-5) while no significant changes were observed in the vaccinated group at any time point.

## Discussion

In this study, potentially protective humoral and cellular immune responses were elicited in mice by immunization using a recombinant adenoviral-based vaccine encoding the S1 subunit of the MERS-CoV spike gene. Several studies have been published that constructed similar vaccines against MERS-CoV and tested their ability to induce production of neutralizing antibodies and other immune system components [13,14,17,19-23]. The results of these studies were encouraging and showed that such vaccines may be promising to prevent the global threat of MERS-CoV infection [13,14,17,19-23]. However, the safety and efficacy of these vaccines still to be confirmed. It was confirmed that the S protein is responsible for the production of neutralizing antibodies [22,31]. The S1 subunit contains the RBD, which binds to its host receptor DPP4 (also known as CD26) and induces production of specific neutralizing antibodies. Several studies have supported the role of this domain to elicit protective immunity against MERS-CoV challenge [25,36,37].

In this study, we were able to demonstrate the distribution and expression of the S1 protein in the vaccinated group but not in the control groups (Ad-CMV and PBS) by detection of the truncated protein-encoding segment in kidney and lung tissues following vaccination using conventional PCR and IHC. These findings support the ability of this vaccine candidate to distribute effectively within host tissues and express the target protein. It has been established that DPP4 is expressed on multiple cell types in the human body including cells in the kidneys and lungs [24]. However, the N glycoprotein, but not the S glycoprotein, of MERS was the target of investigation of MERS-CoV localization using IHC [38]. The N glycoprotein was clearly present in lung tissues following MERS exposure. In fact, the current study can be considered pioneering given the use of the S1 glycoprotein to express viral distribution in tissues. Therefore, the expression of S1 protein in kidney tissues and not in lung might be used to explain previous *in vitro* experiments, which found a cytopathic effect of the MERS-CoV infection inside primary human kidney cells but not in primary human bronchial epithelial cells [39]. In a recent study, the adenoviral vector with enhanced green fluorescent protein but not the adenoviral vector with MERS in a hDPP4 mice experiment after intramuscular or intranasal injection [21]. This supporting our finding that this construct was able to reach the lung tissues, but the S1 protein was not expressed in lung tissues. However, S1 gene detection in lung tissues by conventional PCR and not by IHC indicates further investigation is necessary regarding the level and intensity of S1 expression in this tissue. Additional studies are also needed to explore S1 protein expression in other tissues such as the liver and intestine given the expression of DPP4 in these tissues [40].

In this study, there was a significant serum level of the S1-specific IgG antibody in the vaccinated group but not in the control groups (Ad-CMV and PBS). Similar results have been shown previously: The SARS-CoV vaccine had a strong ability to elicit the S1-specific IgG antibody response [26]. The results presented in this study also supported the findings of recent publications applying a similar vector vaccine construction [13,14,17,19-23].

The T helper 1 immune response is known as the dominant immune response in the case of intracellular pathogens, such as viruses and bacteria, while the T helper 2 immune response is the dominant immune response in cases of extracellular pathogens and allergic reactions [41]. Many pathogens, especially viruses, shift the host immune response toward Th2 dominance over the Th1 response to evade cellular immunity. In this study, Ad-MERS-S1 was able to provoke pro-inflammatory but not anti-inflammatory cytokine release. Both Th1 and Th2 responses were elicited following immunization. Cytokines such as IFN-γ and IL-12, which represent the Th-1 response, were upregulated, while IL-4, which represents the Th-2 response, was downregulated.

The Ad-MERS-S1 was able to induce production of a significant amount of IFN-γ regardless of antigen-specific stimulation. A similar increase in IL-12 was apparent in vaccinated animals in response to specific *in vitro* antigen stimulation. The mRNA expression of IFN-γ was detected as early as 4 weeks after the first immunization. In contrast, IL-4 production in cell culture showed a significant increase in control animals after antigen stimulation, while the production of this cytokine was significantly decreased in the vaccinated group. The results demonstrated neither positive nor negative impact on IL-4 expression in mice vaccinated with the Ad-MERS-S1 or mice that received only PBS. Interestingly, there was a significant increase in IL-4 and IFN-γ gene expression in antigen-stimulated cell culture obtained from mice vaccinated with Ad-CMV at week 6. This might be due to the ability of adenoviruses to elicit strong humoral and cellular immune responses [42,43]. Therefore, it can be concluded that the presence of the S1 protein gene in the adenovirus vector genome disturbs the immune stimulation ability of the adenovirus vector to elicit IL-4 cytokine.

Two studies have shed light on the ability of the S1 subunit, and specifically, the ability of the RBD domain, to shift the host immune response toward Th1 [14,44]. It was demonstrated in these two studies that IgG1, which is produced mainly during the Th2 immune response, was decreased, while IgG2a, which is produced mainly during the Th1 immune response, was upregulated following vaccination with their vaccine candidate. The truncated RBD fused with the Fc portion of human IgG was able to elicit both isotypes with a relatively higher amount of the IgG2a isotype (Th1 response) [44]. Recombinant adenovirus vector carrying the S1 subunit or the whole S gene was also able to induce both types of the immune response. The IgG2a level was detected earlier and higher in MERS-S than MERS-S1 at week 2 post-vaccination but not after week 3 [14]. Accordingly, these findings lead to the dominance toward the Th1 cellular immunity.

Several *in vivo* and *in vitro* studies have revealed the ability of MERS-CoV to evade the host innate immune response through downregulating the antigen-presenting pathway and proinflammatory cytokines such as IFN-γ and IL-12 [38,45-47].

Several MERS-CoV proteins were previously identified as potent IFN antagonists, including the M structural protein and the ORF 4a, ORF 4b, and ORF 5 accessory proteins [46]. In addition, a comparison of the immune response of two MERS-infected patients [48], one of whom with a poor outcome (died) that had elevated levels of IL-17 and IL-10 which promote the Th2 immune response, while the other patient who overcame the infection had increased levels of IFN-α, IFN-γ, and IL-12, which promote the Th1 immune response [48].

In general, it is crucial to induce early and strong innate immune responses against MERS-CoV infection to save lives. Our vaccine candidate was able to induce production of the key cytokines of activated T lymphocytes toward CD4+ Th1 cells, which is also an indication of upregulation of the antigen-presenting pathway. The vaccine also did not trigger the production of IL-4, which is involved in the Th2 response, which may be referred to the counter effect of the produced IFN-γ in IL-4 producing cells [41].

Finally, it is important to clarify that the *in vitro* antigen stimulation was done using the specific 9-mer peptide “CYSSLILDY,” this peptide is located at position 437-445 within the conserved region of the S glycoprotein among all identified MERS-CoV strains. This was selected in a computerized simulation that showed that this sequence has the highest B cell antigenicity plot and has the ability to form the greatest number of interactions with MHC-Class I alleles [33]. Although this epitope was not able to increase IFN-γ production level after *in vitro* stimulation, it resulted in a significant increase in IL-12 production and a significant decrease in IL-4 production in the vaccinated group. In addition, this epitope was able to increase IL-4 production after *in vitro* stimulation in Ad-CMV group. However, these findings were not enough to prove the claim that this epitope maybe has a crucial role in MERS-CoV pathogenesis. Hence, further studies (such as *in vivo* or knockout experiments) should be conducted to address the role of this epitope in MERS-CoV pathogenesis.

## Conclusion

Overall, the adenoviral vector encoding the S1 subunit of the MERS-CoV genome is a promising vaccine candidate against MERS-CoV infection. It was clearly demonstrated that this recombinant vaccine is distributed in host tissues. In addition, this construct could induce production of a specific anti-S1 IgG antibody response as well as the Th1 immune response, which was evident from the increased levels of pro-inflammatory cytokines in the vaccinated animals.

## Authors’ Contributions

MA and MuA designed the adenovirus vaccine for the MERS-S1 vaccine. MA and MK designed the mice experiments. MuA, MA, and MK took the different tissue samples from the mice. MuA performed the molecular assays and the immunohistochemical staining. MA and MuA prepared the manuscript. All authors read and approved the final manuscript.
